# Innervation patterns of sea otter (*Enhydra lutris*) mystacial follicle-sinus complexes

**DOI:** 10.3389/fnana.2014.00121

**Published:** 2014-10-29

**Authors:** Christopher D. Marshall, Kelly Rozas, Brian Kot, Verena A. Gill

**Affiliations:** ^1^Department of Marine Biology, Texas A&M UniversityGalveston, TX, USA; ^2^Department of Wildlife and Fisheries Sciences, Texas A&M UniversityTX, USA; ^3^Marine Mammals Management, U.S. Fish and Wildlife ServiceAnchorage, Alaska, USA

**Keywords:** somatosensory system, peripheral nervous system, axon investment, vibrissae, F-SCs, comparative neurobiology, marine mammals, otters

## Abstract

Sea otters (*Enhydra lutris*) are the most recent group of mammals to return to the sea, and may exemplify divergent somatosensory tactile systems among mammals. Therefore, we quantified the mystacial vibrissal array of sea otters and histologically processed follicle-sinus complexes (F - SCs) to test the hypotheses that the number of myelinated axons per F - SC is greater than that found for terrestrial mammalian vibrissae and that their organization and microstructure converge with those of pinniped vibrissae. A mean of 120.5 vibrissae were arranged rostrally on a broad, blunt muzzle in 7–8 rows and 9–13 columns. The F-SCs of sea otters are tripartite in their organization and similar in microstructure to pinnipeds rather than terrestrial species. Each F-SC was innervated by a mean 1339 ± 408.3 axons. Innervation to the entire mystacial vibrissal array was estimated at 161,313 axons. Our data support the hypothesis that the disproportionate expansion of the coronal gyrus in somatosensory cortex of sea otters is related to the high innervation investment of the mystacial vibrissal array, and that quantifying innervation investment is a good proxy for tactile sensitivity. We predict that the tactile performance of sea otter mystacial vibrissae is comparable to that of harbor seals, sea lions and walruses.

Sea otters (*Enhydra lutris*) are the smallest and most recent group of marine mammals to return to marine habitats. They are thought to have arisen in the North Pacific during the Pleistocene (Leffler, 1964; Mitchell, 1966; Repenning, 1976) and have only become fully aquatic in the last 1–3 million years (Berta and Sumich, 1999). Historically, sea otters occurred in near shore waters around the North Pacific rim from Hokkaido, Japan through the marine coastal areas of the Russian Far East and the Pacific coastal areas in the United States as far south as Baja California (Japan; Kenyon, 1975; Riedman and Estes, 1990). Three subspecies are recognized throughout this wide range: Southern sea otters (*E.l. nereis*), Northern sea otters (*E.l. kenyoni*), and Russian sea otters (*E.l. lutris*; Kenyon, 1975; Riedman and Estes, 1990). Sea otters play an important ecological role in structuring near shore benthic communities upon which they inhabit and forage (Estes and Palmisano, 1974; Estes and Duggins, 1995). Tactile senses are likely important during foraging dives in which they dig for infaunal bivalves, collect epifaunal invertebrates, or capture cephalopods and occasionally several species of fishes near the sea-floor (Kenyon, 1975; Kvitek et al., 1988; Riedman and Estes, 1990). In general, the foraging ecology of all otters (Carnivora: Mustelidae: Lutrinae) is often categorized as either piscivorous mouth-oriented or invertebrate hand-oriented predation (Radinsky, 1968; Duplaix, 1984; Sivasothi and Nor, 1994; Jacques et al., 2009; Timm, 2013). These foraging categories manifest themselves as divergent feeding kinematics, biomechanics and performance (Timm, 2013) and were originally based on brain sulcal patterns (Radinsky, 1968) and morphological characters (Van Zyll de Jong, 1972).

Sea otters use their hands, digits and vibrissae to explore their environment, likely to assist them in identifying prey items. Vibrissae are specialized hair structures comprised of a hair-shaft surrounded by several blood-filled sinuses and technically termed a Follicle-Sinus Capsule (F - SC; Figure 2). Most of our knowledge regarding F - SC structure and function is based on terrestrial taxa (Davidson and Hardy, 1952; Melaragno and Montagna, 1953; Andres, 1966; Burgess and Perl, 1973; Gottschaldt et al., 1973; Halata, 1975, 1993; Halata and Munger, 1980; Rice et al., 1986, 1993, 1997; Brecht et al., 1997; Ebara et al., 2002), in which they are functionally divergent from other types of hair. Among aquatic mammals, afferent fibers from F-SCs respond best to vibrotactile stimuli (Dykes, 1974), but are used for both active-touch discrimination and hydrodynamic reception in pinnipeds (Stephens et al., 1973; Ling, 1977; Marshall et al., 2006; Hanke et al., 2013) and sirenians (Reep et al., 1998, 2001, 2002, 2011; Gaspard et al., 2013). Pinnipeds are known to possess the largest mystacial F-SCs (Ling, 1977). The hair shafts (HS) of many pinniped vibrissae are beaded in their appearance and show variation in this bumpy pattern (Ginter et al., 2010, 2012). This phenotype has been shown to decrease turbulent water flow over the HS (reviewed by Hanke et al., 2013). Furthermore, pinniped F-SCs diverge from terrestrial mammals in numerous ways, but most prominently in that they (1) possess a tripartite blood sinus system vs. a bipartite blood sinus system; (2) lack a superficial vibrissal nerve that innervates the apical regions of the F - SC; (3) possess a deep vibrissal nerve (DVN) that provides innervation to the entire F - SC and penetrates the external collagenous dermal capsule (DC) at the base of the follicle as opposed to entering the DC laterally near the lower cavernous sinus (LCS) and ring sinus (RS); (4) possess a greater number of axons per F - SC (~1000–1600 in pinnipeds vs. ~200 in other mammals; Yablokov and Klezeval, 1969; Hyvärinen and Katajisto, 1984; Hyvärinen, 1989, 1995; Marshall et al., 2006). The F-SCs of sea otters remain unexplored, but as mammals that have recently returned to the sea, the functional and neural changes in vibrissal structure of otters during this transition are of evolutionary interest.

**Figure 1:**
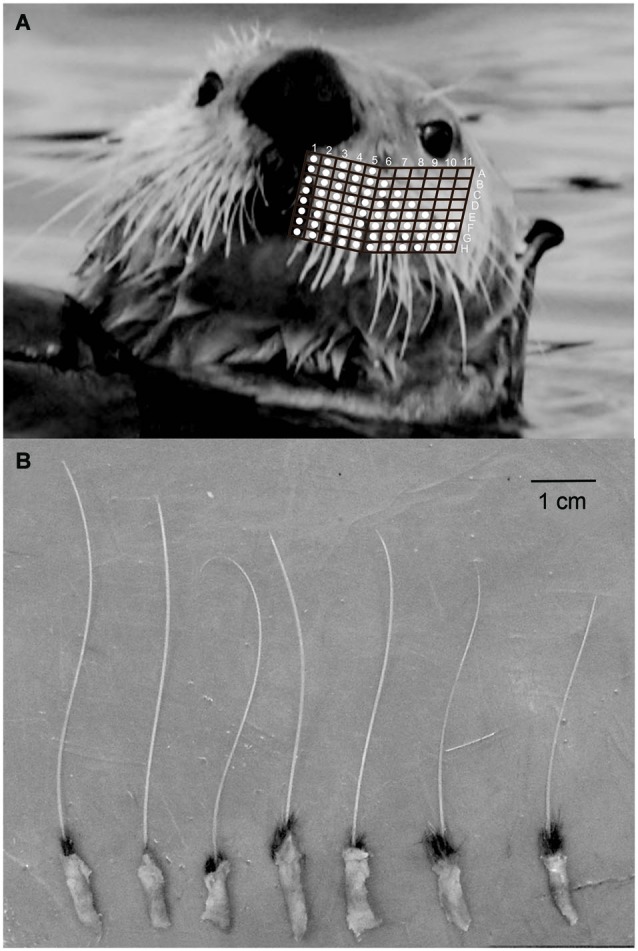
**(A)** Sea otter mystacial vibrissae *in vivo* with overlain representative map of columns and rows of individual vibrissa. (**B)** Isolated mystacial vibrissae from rows G-H, columns 9–11.

**Figure 2:**
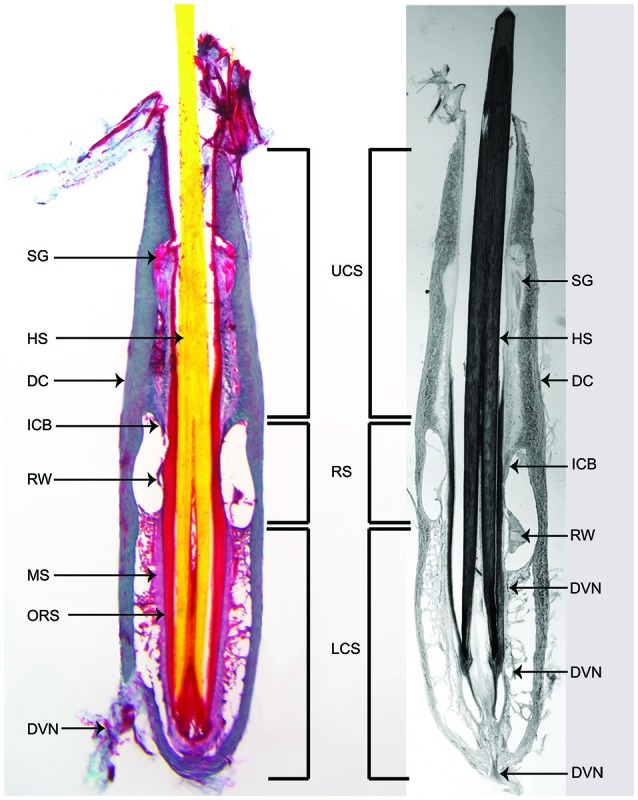
**Longitudinal sections through the center of large mystacial F-SCs stained with trichrome (left) and a modified Bodian silver stain (right) for axons**. UCS: upper cavernous sinus; RS: ring sinus; LCS: lower cavernous sinus; SG: sebaceous gland; HS: hair shaft; DC: dermal capsule of follicle: ICB: inner conical body; RW: ringwulst; MS: mesenchymal sheath; ORS: outer root sheath; DVN: deep vibrissal nerve. *Note the penetration of the DVN at the base of the F - SC and its route through the LCS to the RW*.

In lieu of comparable cortical and electrophysiological for sea otters, quantification of sea otter F - SC innervation can serve as a proxy for performance (George and Holliday, 2013). Quantifying the innervation of sea otter vibrissae can highlight somatosensory constraints and selection pressures of an aquatic environment. Is sea otter mystacial vibrissae morphology similar to terrestrial taxa, pinnipeds, a hybrid of both, or does it represent a completely novel microstructure and innervation? Due to their expanded vibrissal apparatus, we hypothesized that (1) the number of myelinated axons per F - SC, is greater than terrestrial mammalian vibrissae; and that (2) the microstructure of sea otter F-SCs converge with the vibrissae of pinnipeds rather than with those of terrestrial mammals, or a *de novo* organization.

## Materials and methods

Whole bilateral mystacial vibrissal arrays (*N* = 6) of sea otters were collected during necropsies at the U.S. Fish and Wildlife Service’s Marine Mammals laboratory in Anchorage, AK (USA). Samples were immersed in 10% phosphate buffered formaldehyde. Mystacial vibrissal arrays were photographed and individual whiskers were counted and mapped (Figure 1). Lengths and diameters of the largest six individual vibrissae from each specimen were measured with digital calipers (*n* = 24). Sea otter F-SCs are relatively small, so six of the largest mystacial F-SCs were dissected from each specimen (*n* = 36) and frozen-sectioned either in the longitudinal plane or in cross-section using a Lipshaw 80A sliding stage microtome fitted with a circulating water freezing stage (Physitemp Instruments, Clifton, NJ) at 30 µm. Longitudinal sections and cross-sections were used for both neural and microstructural analyses. Sections were stained with either a modified Masson’s trichrome stain or a modified Bodian silver stain (Armed Forces Institute of Pathology, 1968, 1994; Reep et al., 2001, 2002; Marshall et al., 2006). Histochemical stains were used because it was not possible to collect samples from remote locations in a manner that would have allowed immunolabeling techniques (e.g., Rice et al., 1997; Ebara et al., 2002; Sarko et al., 2007).

The following morphometric data were collected from histologically processed longitudinal sections: maximum F - SC length, maximum total sinus length (upper cavernous sinus [UCS] + ring sinus [RS] + lower cavernous sinus [LCS]), maximum RS width, maximum DC thickness, and maximum HS diameter at the level of the RS. The following morphometrics were collected from histologically processed cross-sections: maximum diameter of section (including DC), maximum longitudinal axis diameter of the HS, maximum perpendicular axis diameter of HS, DC thickness, and mesenchymal sheath (MS) thickness.

F - SC innervation patterns, myelinated axon counts, and identification of mechanoreceptor location were investigated using longitudinal sections and cross-sections stained with a modified Bodian silver stain, but also a trichrome stain. Myelinated axons were identified in cross-sections by their myelinated sheaths and were quantified midway along the length of the LCS following Marshall et al. (2006) and McGovern et al. (accepted). Axons were counted at this location because axon numbers decrease above this location as the DVN ascends toward the RS (Rice et al., 1986). Conducting counts in this location is consistent with other studies, allowing a comparison of axon counts among species. Mystacial vibrissal innervation was quantified by (1) summing the axons in each cross-section; (2) averaging the axons from a minimum of four cross-sections from each F - SC from four individuals; (3) calculating the mean number of axons/F - SC from all six individuals; (4) calculating the mean number of vibrissae per vibrissal array; and (5) calculating the mean total innervation to the mystacial vibrissal array by multiplying the overall mean number of axons/F - SC by the mean number of vibrissae. Where possible we investigated the location of mechanoreceptors, but further characterization of sea otter mechanoreceptors was beyond the scope of this study.

## Results

The mean number of sea otter mystacial vibrissae was 120.5 (±7.29), which were arranged in 7–8 rows and 9–13 columns (Figure 1). Mystacial vibrissae are organized as macro- and micro-vibrissae and were located laterally and medially, respectively, within each mystacial array. The mean length of the largest mystacial vibrissal HS was 7.13 ± 1.60 mm, but wear on all HS was evident. Vibrissal HS were circular in cross-section. The ratio of two hair-shaft diameters 90° to each other was 0.97 ± 6.5 and the ratio from histologically processed cross-sections was 1.00 (±0.04; Table 1). The HS profiles were smooth, that is they did not exhibit any beaded appearance.

**Table 1:** **Cross-sectional F - SC morphometrics**.

	Mean	S.D.	Minimum	Maximum
Max. diameter (mm)	2.1	0.20	1.0	2.4
Mean longitudinal axis of HS (mm)	0.5	0.08	0.4	0.7
Mean perpendicular axis of HS (mm)	0.5	0.08	0.4	0.7
Ratio HS diameter	1.0	0.04	0.9	1.1
Mean DC thickness (mm)	0.2	0.04	0.1	0.3
Mean MS thickness (mm)	0.1	0.04	0.1	0.3
Mean CT thickness (mm)	0.3	0.05	0.1	0.4

*N = 25*.

**Figure 3:**
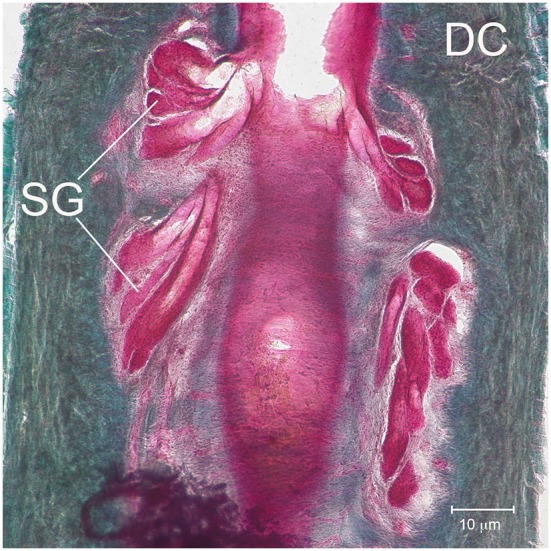
**Sebaceous glands**. Detailed micrograph of SG near the apical region of the UCS and surrounded by the apical DC.

Internally the F-SCs of sea otters exhibited a tripartite organization. Each F - SC was comprised of an UCS, RS, and a LCS (Figure 2). The UCS comprised ~40% of the total F - SC length (Table 2). A relatively sizable Ringwulst (RW) was present in the RS (Figure 2). Thin collagenous trabeculae spanned both the UCS and the LCS, but not the RS. Sebaceous glands were located within the walls of the UCS, but no other types of glands were observed (Figures 2, 3).

**Table 2:** **Longitudinal F - SC morphometrics**.

	Mean	S.D.	Minimum	Maximum
Mean max F - SC length (mm)	9.9	1.24	7.8	12.3
Mean max. UCS length (mm)	2.9	0.31	2.2	3.6
Mean max. RS length (mm)	1.8	0.21	1.3	2.1
Total sinus length (mm)	7.8	0.65	6.3	8.5
Mean max. LCS length (mm)	3.5	0.52	2.5	5.3
% UCS length to total F - SC length	39.4	3.19	33.7	45.9
Mean max. RS width (mm)	0.5	0.09	0.3	0.7
Mean max. DC thickness (mm)	0.4	0.07	0.16	0.5

*N = 22*.

**Figure 4:**
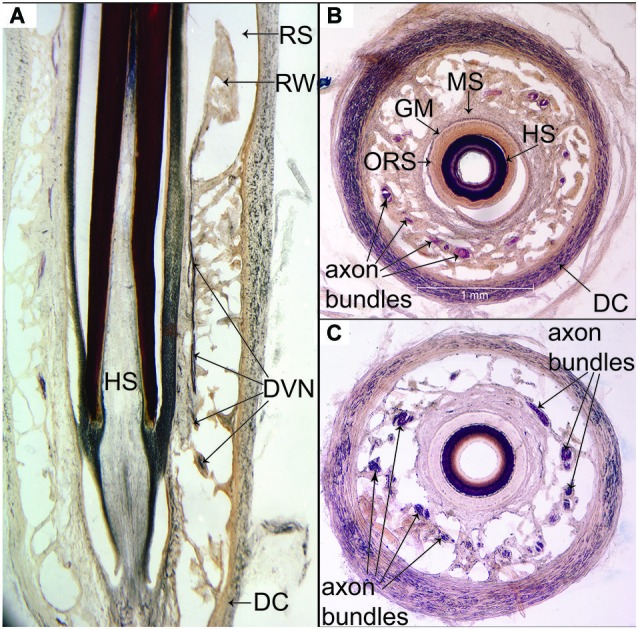
**Silver staining of sea otter F-SCs. (A)** Longitudinal center section depicting the DVN ascending from the base of the LCS through the trabeculae and the MS to the RW within the RS. **(B)** Cross-section midway through the LCS showing the location and relative concentric organization of the DC, axon bundles within the trabeculae, MS, GM (glassy membrane), ORS and HS. **(C)** Silver stained axon bundles distributed throughout the trabeculae of the LCS.

Each F - SC was innervated by a DVN (Figures 2, 4), a branch of the infraorbital branch of the trigeminal nerve (CN V). The DVN penetrated the DC at, or near, the basal end of the F - SC. Upon entering the F - SC the DVN coursed apically, and branched into major bundles throughout the mid-UCS to innervate structures in the LCS, RS, RW and inner conical body (ICB). No innervation to the UCS by superficial vibrissal nerves, or other nerves, was observed. The mean number of myelinated axons innervating each F - SC was 1339 ± 408.3 axons. Since sea otters have a mean number of 120.5 vibrissae, the mean number of myelinated axons estimated to innervate the mystacial vibrissal array was 161,313.

**Figure 5:**
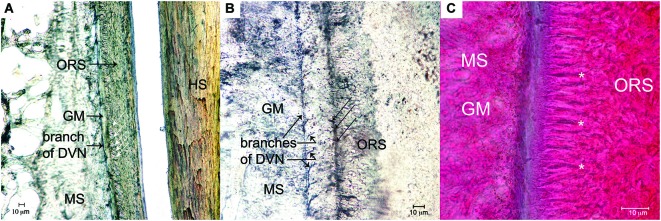
**Mechanoreceptors of the F - SC. (A)** Longitudinal branch of the DVN coursing apically within the MS, but adjacent to the GM. White arrows show the location of presumptive Merkel-Neurite Complexes (MNCs) within the ORS adjacent to the GM (Bodian silver, 20X). **(B)** Longitudinal branch of the DVN coursing apically in the MS with numerous sub-branches crossing the GM to innervate presumptive lanceolate mechanoreceptors (black arrows) in the ORS (Bodian silver stain 40X). **(C)** Location of presumptive mechanoreceptors (asterisks) arranged in the ORS at the border of the GM (trichrome, 100X).

Mechanoreceptors were observed from the mid-LCS to the RS and ICB. Along its course the DVN, fibers were observed to branch off and course parallel to the glassy membrane (GM) and the MS (Figure 5A). Numerous branches were observed to leave these fibers and cross the GM and to supply presumptive mechanoreceptors in the outer root sheath (ORS), which appear to be Merkel-Neurite Complexes and lanceolate mechanoreceptors (Figure 5B). Mechanoreceptor type requires further confirmation using transmitted electron microscopy. Presumptive mechanoreceptors were always observed at the boundary of the GM and ORS (Figure 5C) from the mid-LCS, to the RS and ICB.

## Discussion

Sea otters possess a bilateral mystacial vibrissal array that is oriented rostrally on a broad and blunt muzzle. This is in contrast to many pinnipeds that feed in the water column and possess more laterally placed mystacial vibrissal arrays. The forward orientation of sea otter vibrissae is ideal to interact with, and assist in identifying, epibenthic and infaunal prey. The mean number of individual mystacial vibrissae in sea otters reported here is consistent with numbers reported by Kenyon (1975) and the rostral orientation of mystacial vibrissae on the rostrum is consistent with other benthic foragers such as bearded seals (*Erignathus barbatus*; Marshall et al., 2006) and walruses (Fay, 1982; Kastelein and Mosterd, 1989; Marshall et al., 2006, 2008). Sea otters possess fewer vibrissae than bearded seals (Marshall et al., 2006) and walruses (*Odobenus rosmarus*; Fay, 1982), which is not surprising considering their small body size. Sea otter hairshafts lacked a beaded profile, and the circular cross-section is consistent with active touch performance rather than hydrodynamic reception, similar to these other benthic foragers. Sea otter F-SCs are similar to pinnipeds in that they are organized in a tripartite blood sinus system and possess common traits that pinnipeds exhibit, such as penetration of the DVN at the base of the follicle. In addition, the UCS of sea otters is relatively long and their UCS lacks innervation (i.e., a superficial vibrissal nerve as observed in terrestrial species). Although the long UCS of sea otters is similar to pinnipeds, it is relatively shorter than harbor seals (*Phoca vitulina*) and bearded seals, which are hypothesized to thermally protect the sensitive mechanoreceptors within the ICB, RS, and LCS from cold water (Dehnhardt et al., 1998; Mauck et al., 2000; Marshall et al., 2006; Erdsack et al., 2014). We propose a similar function in sea otters, which would also be advantageous for a small marine mammal with an elevated metabolic rate (Riedman and Estes, 1990; Yeates et al., 2007). Although the relative length of the UCS of sea otters is not as great as observed in harbor and bearded seals, sea otters do not experience extremely cold water as do these seals. The total innervation to the mystacial vibrissal array of sea otters (161,313 axons) is ~50% less than bearded seals (320,616; Marshall et al., 2006), but this is only due to the fewer number of mystacial vibrissae, not a reduction in the number of axons/F - SC. The innervation estimate to the entire mystacial vibrissal array in sea otters is nearly the same as ringed seals (*Pusa hispida*, 160,000; Hyvärinen et al., 2009) and northern elephant seals (*Mirounga angustirostris*, ~159,000; McGovern et al., accepted). It should be noted that such estimates of innervation to the entire mystacial array may be overestimated since smaller F-SCs may have fewer axons/F - SC as found in rodents (Lee and Woolsey, 1975; Welker and Van der Loos, 1986). However these relative measures are useful in a comparative context until more specific relationships between F - SC size and innervation become available for aquatic mammals. Overall, these results show that despite the recent return to the marine environment by sea otters, their F - SC microstructure and innervation have converged with their pinniped counterparts (Stephens et al., 1973; Hyvärinen and Katajisto, 1984; Hyvärinen, 1989, 1995; Marshall et al., 2006; Dehnhardt and Mauck, 2008; Hyvärinen et al., 2009). The selection pressures for an increased tactile capability for aquatic and benthic foraging likely resulted in the increased innervation of sea otter mystacial vibrissae compared to terrestrial mammals.

A comparative brain endocast study of sulcal patterns in all extant genera of otters by Radinsky (1968) reported two specializations of somatosensory cortex that relate to their tactile discrimination capabilities. Specifically, the coronal gyrus is enlarged in all otter genera relative to terrestrial carnivores. In addition, the brains of sea otters and clawless otters (*Aonyx sp*., including members formally in the genus *Amblonyx* and *Paraonyx*; Bininda-Emonds et al. (1999) and Wilson and Reeder (2005)) also exhibit an expanded postcruciate gyrus. Although no electrophysiological mapping data of otters are available, electrophysiological mapping of the coronal gyrus in canids, felids, procyonids (Pinto Hamuy et al., 1956; Woolsey, 1958; Welker and Seidenstein, 1959; Welker and Campos, 1963; Welker et al., 1964), as well as northern fur seals (*Callorhinus ursinus*; Ladygina et al., 1992) demonstrate that this cortical region receives somatosensory projections from the head, and particularly from vibrissae. Furthermore, in Northern fur seals, afferent fibers from each mystacial vibrissa project to isolated and non-overlapping somatosensory cortex in the coronal gyrus (Ladygina et al., 1992). This cortical region in Northern fur seals is greatly expanded relative to the representation of other parts of the head, and is an example of cortical magnification found in somatosensory systems in a variety of specialized vertebrates (Catania, 2007). Likewise, the postcruciate gyrus receives somatosensory afferents from the forelimb (Pinto Hamuy et al., 1956; Woolsey, 1958; Welker and Seidenstein, 1959; Welker and Campos, 1963; Welker et al., 1964). These relationships appear to be a generalized pattern of mammalian brain organization.

Our data show that sea otter F - SC innervation supports Radinsky’s hypothesis that the disproportionate expansion of the somatosensory regions in the coronal gyrus presents strong evidence of “an unusually high degree of development” of vibrissal afferents. Using comparative inference, the axons of sea otter F-SCs should project to the coronal gyrus in somatosensory cortex as observed in canids, felids, procyonids, and fur seals (Pinto Hamuy et al., 1956; Woolsey, 1958; Welker and Seidenstein, 1959; Welker and Campos, 1963; Welker et al., 1964; Ladygina et al., 1992). Such inferences are commonly used in paleoneuroanatomy and brain endocast data have been used to investigate the evolution of cortical regions in extinct and extant canids (e.g., Radinsky, 1967, 1968, 1973; Finarelli and Flynn, 2007; Lyras, 2009). This expansion of vibrissal innervation and corresponding somatosensory cortex also suggests increased tactile sensitivity to the vibrissae for all otters, as well as increased tactile sensitivity of the forelimbs for members of the genus *Enhydra* and *Aonyx* (Radinsky, 1968).

The use of mystacial vibrissae by pinnipeds to explore their environment is well known. Among pinnipeds, vibrissal function is perhaps best known for harbor seals (*Phoca vitulina*; reviewed by Hanke et al., 2013) and California sea lions (*Zalophus californianus*; Dehnhardt and Dücker, 1996). The lateral macro-vibrissae of pinnipeds are used to explore large-scale environmental features and their medial micro-vibrissae are used for more refined and discrete tactile exploration (Dehnhardt and Kaminski, 1995; Dehnhardt and Dücker, 1996; Grant et al., 2013; Marshall et al., 2014). The resolution of tactile sensitivity, as characterized by a dimensionless Weber fraction, for harbor seals and California sea lions (*Zalophus californianus*) is approximately the same as for mammals using prehensile organs or monkeys using their hands (Dehnhardt, 1994; Dehnhardt and Kaminski, 1995; Dehnhardt and Dücker, 1996). Although it is less well known, it is clear that walruses can detect differences in surface area as small as 0.4 cm^2^ with their mystacial vibrissae, which is likely sensitive enough to distinguish among similar-sized prey types (Kastelein and van Gaalen, 1988). Based on the similarities of sea otter F-SCs in organization, innervation pattern, and presumptive projection of these axons to the coronal gyrus to somatosensory cortex as in pinnipeds, and electrophysiological mapping of fur seals and other mammalian taxa, we predict that the tactile performance of sea otter mystacial vibrissae should be at the level of harbor seals and California sea lions. Psychophysical testing of sea otter whisker sensitivity should be conducted to test this hypothesis. Clearly further investigation of the coronal, postcruciate and somatosensory cortical neuroanatomy and function are needed to further explore tactile performance in otters, as well as detailed data regarding these peripheral mechanoreceptors and associated structures.

Although much more is known regarding the innervation and function of terrestrial mammalian vibrissae, much of this knowledge is based on laboratory animal species. The limited comparative data regarding aquatic mammal vibrissae innervation indicate that aquatic and semi-aquatic mammals possess a greater innervation investment than terrestrial mammals (Hyvärinen, 1989, 1995; Hyvärinen et al., 2009). The F-SCs of rodents, rabbits, cats, and monkeys are innervated by approximately 100–200 myelinated axons (Halata, 1975; Lee and Woolsey, 1975; Halata and Munger, 1980; Rice et al., 1986, 1997; Ebara et al., 2002). This number is consistent for most terrestrial mammals. Pinniped innervation investment ranges 1000 to 1600 axons/F - SC. However, a more direct within Family aquatic vs. terrestrial comparison not only supports this pattern, but also now suggests a hierarchy of higher to lower innervation from fully- to semiaquatic to terrestrial species. For example, Australian water rats (a semi-aquatic species) possess 500 axons/F - SC (*Hydromys crysogaster*; Dehnhardt et al., 1999) compared to laboratory rats (~100 axons/F - SC), a terrestrial species. The number of axons/F - SC of sea otter mystacial vibrissae (1339), a fully-aquatic mustelid, is greater than semi-aquatic European river otters (~500; *Lutra lutra*; Hyvärinen et al., 2009) and polecats (130; *Mustela putorius*), a terrestrial mustelid (Hyvärinen et al., 2009). The innervation of sea otter vibrissae is comparable to phocid seals and fits the fully-aquatic F - SC innervation investment pattern. Sirenians and odontocetes are exceptions to this pattern due to their adaptations for feeding on aquatic plants, and innovation of echolocation, respectively. However, as additional comparative data are collected from novel senses, such as the modification of vibrissae for electroreception in certain river dolphins (Czech-Damal et al., 2012), innervation investment from these senses may also fall within this pattern.

Continued investigations of aquatic mammals, including sea otters, are needed regarding segregation of information processing in trigeminal-thalamic projections to the coronal gyrus and cortical organization related to somatosensory systems. Such work will be important in elucidating aquatic sensory adaptations and the evolution of the mammalian somatosensory nervous system in secondarily aquatic mammals and tetrapods.

## Author contributions

All authors had full access to all the data in the study and take responsibility for the integrity of the data and the accuracy of the data analysis. Study concept and design: Christopher D. Marshall. Acquisition of data: Kelly Rozas, Christopher D. Marshall, Verena A. Gill, Brian Kot. Analysis and interpretation of data: Christopher D. Marshall, Kelly Rozas. Drafting of the manuscript: Christopher D. Marshall. Critical revision of the manuscript for important intellectual content: Christopher D. Marshall, Kelly Rozas, Verena A. Gill, Brian Kot. Obtained funding: Christopher D. Marshall, Verena A. Gill.

## Conflict of interest statement

The authors declare that the research was conducted in the absence of any commercial or financial relationships that could be construed as a potential conflict of interest.

## Abbreviations

CT: Collagenous Trabeculae
DC: Dermal Capsule
DVN: Deep Vibrissal Nerve
F - SC: Follicle-Sinus Complex
GM: Glassy Membrane
HS: Hair Shaft
ICB: Inner Conical Body
LCS: Lower Cavernous Sinus
MNC: Merkel-Neurite Complex
MS: Mesenchymal Sheath
ORS: Outer Root Sheath
RS: Ring Sinus
RW: Ringwulst
SG: Sebaceous Gland
UCS: Upper Cavernous Sinus.
